# The Lack of Predictors for Rapid Progression in Prostate Cancer Patients Receiving Sipuleucel-T

**DOI:** 10.3390/cancers5020511

**Published:** 2013-05-06

**Authors:** Laura Ng, Wendy Heck, Stacey Lavsa, David Crowther, Brad Atkinson, Lianchun Xiao, John Araujo

**Affiliations:** 1 Division of Pharmacy, MD Anderson Cancer Center, 1515 Holcombe Blvd Houston, TX 77030, USA; E-Mails: laurangpharmd@gmail.com (L.N.); wdsmith@mdanderson.org (W.H.); smlavsa@mdanderson.org (S.L.); dmcrowther@mdanderson.org (D.C.); 2 Division of Biostatistics, MD Anderson Cancer Center, 1515 Holcombe Blvd Houston, TX 77030, USA; E-Mail: lxiao@mdanderson.org; 3 Division of Genitourinary Medical Oncology, MD Anderson Cancer Center, 1515 Holcombe Blvd Houston, TX 77030, USA; E-mail: johna@mdanderson.org

**Keywords:** prostate cancer, sipuleucel-T, immunotherapy, progression, metastasis

## Abstract

Sipuleucel-T is an immunotherapy indicated for the treatment of metastatic prostate cancer. It offers a new mechanism to treat prostate cancer without the side effects of hormone therapies and chemotherapies. In previous studies sipuleucel-T did not delay disease progression, but demonstrated an overall survival benefit compared to placebo. While clinical trials have evaluated the effects of sipuleucel-T on overall survival and progression, more studies are needed to evaluate its effectiveness and role in the management of prostate cancer. The objective of this study is to identify the incidence and possible predictors for disease progression in patients receiving sipuleucel-T. A retrospective review of patients who received sipuleucel-T between 1 September 2010 and 11 October 2011 was conducted (n = 36). Patients who changed therapy or died within 120 days were classified as experiencing rapid progression. Potential predictors of rapid progression were examined using logistic regression. Seven patients met criteria for rapid progression. Progression occurred in 72.2% of all patients. The median days to progression was 158. No significant predictors of rapid progression were identified. Currently no predictors have been found to be associated with rapid progression in prostate cancer patients on sipuleucel-T.

## 1. Introduction

Prostate cancer is the most common cancer diagnosed among men and is the second leading cause of cancer mortality in men in the United States [1]. Initial local therapy can help control disease, but 20–40% of patients will eventually experience recurrence or metastatic disease, most commonly to the bones and/or regional lymph nodes [2]. Metastatic prostate cancer is initially treated with androgen deprivation, which can stabilize or cause regression of disease in up to 85% of patients [3,4]. Despite initial treatment with androgen deprivation therapy and other secondary hormonal agents, all patients ultimately develop castration refractory prostate cancer (CRPC) [2,3,4]. Management of CRPC is clinically challenging because of limited options for therapy. Current treatment options include second-line hormone therapy, chemotherapy or investigational agents [2,4,5,6]. Sipuleucel-T (Provenge^®^, Dendreon Corp.) is a novel therapy that offers a new mechanism for treating metastatic prostate cancer. It is the first FDA-approved immunotherapy for the treatment of asymptomatic or minimally symptomatic metastatic prostate cancer refractory to castration therapy [7].

Sipuleucel-T is an autologous active cellular immunotherapy product engineered to induce a T-cell response against prostatic acid phosphatase (PAP), an antigen expressed in a majority of prostate cancer [4,5,6,7]. The treatment consists of autologous peripheral immune cells including antigen-presenting cells (APCs), which are activated *in vitro* with a recombinant fusion protein known as PA2024 [5,6,7,8,9]. PA2024 is comprised of PAP and granulocyte-macrophage colony-stimulating factor (GM-CSF), an immune cell activator [7,8]. Randomized, placebo-controlled phase III trials have examined the use of sipuleucel-T in prostate cancer patients and demonstrated a survival benefit for patients treated with sipuleucel-T [3,4,10]. None of the trials, however, have demonstrated a difference in time to progression between the patients who received sipuleucel-T and those who received placebo, an infusion of the patient’s own cells that were not activated with PA2024. The Immunotherapy for Prostate Adenocarcinoma Treatment (IMPACT) group conducted the largest phase III trial consisting of 512 patients and found an overall survival benefit in those who received sipuleucel-T over those in the control arm. However, the median time to progression in both groups was the same, approximately 120 days or 4 months [10]. With initial use of sipuleucel-T at our institution, rapid progression was observed in some patients. Therefore we conducted this investigation to identify possible predictors for rapid progression. Although sipuleucel-T has been shown to have a benefit on overall survival, studies evaluating possible factors for rapid progression are needed to help further identify patients who could gain the most from receiving the treatment. As an increasing number of people consider sipuleucel-T as an option for management of prostate cancer, information regarding its use and adverse effects will be valuable to clinical practice.

## 2. Experimental Section

In this retrospective chart review, prostate cancer patients who received at least one dose of sipuleucel-T during the time period of 1 September 2010 and 11 October 2011 were included in the study. Subjects were identified through electronic pharmacy records based on dispensing for sipuleucel-T. The primary objective of this study was to identify the incidence and possible predictors for disease progression comparing rapid progression (RP) and non-rapid progression (NRP). Secondary objectives included comparing the time to progression in the RP and NRP groups as well as examine overall incidence of infusion related reactions.

Progression was defined as a change in therapy for prostate cancer. RP was defined as switching therapy or experiencing death as documented in the electronic database within 120 days of receiving the first dose of sipuleucel-T. The patients’ electronic medical records were accessed to collect data including: patient demographics, Gleason scores, radiographic and laboratory results, Eastern Cooperative Oncology Group (ECOG) performance status, metastatic disease sites, any documented therapies for the treatment of prostate cancer, concomitant analgesics, documented switch in therapy, and infusion-related reactions. Patients were then stratified into RP and NRP prostate cancer groups. Once patients were stratified into the groups, outcome data comparing the two groups was analyzed. Potential predictors included in the analysis were: age, weight, Gleason score, primary Gleason grade, bone metastases, lymph node metastases, analgesic use, previous cancer therapy, prostate-specific antigen (PSA), absolute neutrophil count, white blood cell count, alkaline phosphatase, and lactate dehydrogenase.

For continuous data, summary statistics including sample size, mean, standard deviation, median, minimum and maximum were computed. For discrete or categorical data, descriptive statistics included tabulations of frequencies. Discrete data was evaluated using student’s T-test for continuous variables and Fisher’s exact test for categorical data. To identify independent patient characteristic predictors of rapid progression, a univariate analysis was performed using Statistical Packages for the Social Sciences (SPSS) software. For the univariate analysis, all variables with a p value less than 0.05 were considered statistically significant. All tests were two-sided.

## 3. Results

### 3.1. Baseline Characteristics

From 1 September 2010 and 11 October 2011, 36 prostate cancer patients who received at least one dose of sipuleucel-T were identified. All patients received the full three courses of sipuleucel-T therapy. The median age of the patients at time of sipuleucel-T therapy initiation was 67 years (range 54–81 years). All patients had a good performance status with an ECOG score of 0 or 1. The median Gleason score and primary Gleason grade were 8 and 4, respectively. While 58.3% of patients had either disease involvement of the bone or lymph node, a smaller proportion, 22.2%, had disease involving both the bone and lymph node. At baseline, 36% of patients were using analgesic therapy prior to sipuleucel-T therapy. All patients had been receiving androgen deprivation therapy. A large number of patients were previously treated with antiandrogren therapy (94.4% of patients), with more than half of the entire group also having been previously treated with a second line anti-hormonal agent. A majority of patients had a radical prostatectomy. More than half of all patients received radiation to the prostate or the prostate bed. Thirty percent of patients were previously treated with chemotherapy, with more than half of those patients receiving docetaxel. The median prostate-specific antigen (PSA) level was detectable at 6.4 nanograms/milliliter. A detailed account of patient baseline characteristics can be found in Table 1.

**Table 1:** Patient baseline characteristics (n = 36).

Patient characteristics	
Median age, years (range)	67 (54–81)
Race (%)	
Caucasian	91.6
African American	5.6
Other	2.8
ECOG performance status 0 or 1 (%)	100
Median weight in kilograms (range)	89.9 (70.2–177.9)
Median Gleason score	8
Median Primary Gleason grade	4
Disease location (%)	
Bone	58.3
Lymph node	58.3
Both	22.2
Analgesic use (%)	36
Previous cancer therapy (%)	
Androgen deprivation therapy	100
Antiandrogen	94.4
Second line anti-hormonal agent	55.6
Radical prostatectomy	74.3
Radiation to prostate or prostate bed	58.3
Chemotherapy	30.6
Docetaxel	16.7
Median laboratory values	
PSA (ng/mL)	6.4
Alkaline phosphatase^+^ (units/L)	69
Lactate dehydrogenase (units/L)	452.5
White blood cell count (cells/mm^3^)	6.8
Absolute neutrophil count (cells/mm^3^)	3.8

^+^ The normal range for alkaline phosphatase is 31 to 131 units per liter, depending on age.

### 3.2. Post Sipuleucel-T Therapy

**Figure 1:**
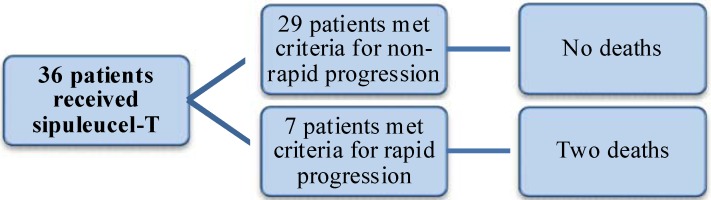
Delineation of rapid progression group and non-rapid progression groups.

A majority of patients progressed after receiving sipuleucel-T therapy (72.2%). Almost 20% of patients (n = 7) had switched therapy within 120 days and therefore met criteria for the RP group. Two deaths occurred in the rapid progression group and none at the time of analysis in the NRP group. Figure 1 demonstrates the delineation between both groups.

For all 36 patients, the median days to progression as defined by a switch in therapy, was 158 days (range 71–367 days). The median days to progression was 104 in the RP group and 168 in the NRP group (Table 2). The overall incidence of infusion-related reaction in the group was 8.3%, with a majority of the reactions manifesting as fever and chills. No cerebral events were found.

**Table 2:** Progression and infusion-related reactions in patients after receiving sipuleucel-T.

Progression and infusion-related reactions	
Progression (%)	72.2
Rapid Progression (%)	19.4
Median days to progression (range)	
Overall	158 (71–367)
Rapid progression group	104 (71–111)
Non-rapid progression group	168 (124–367)
Infusion-related reaction (%)	8.3

### 3.3. Univariate Analysis for Risk Factors

No significant differences in risk factors were found between the RP and NRP groups after sipuleucel-T treatment (Table 3). There was a difference between the RP and NRP groups in use of second line anti-hormonal agents, but this difference was not found to be significant. There was also no significant difference found between the RP or NRP groups in other previous cancer therapy. Although there was difference in median baseline PSA between the two groups, this was not found to be significant. 

**Table 3:** Univariate analysis of risk factors.

Patient characteristics	RP (n = 7)	NRP (n = 29)	*p*-value
Median age, years	70	67	0.128
Median weight (kilograms)	89.8	90	0.890
Median Gleason score	7	8	0.190
Median Primary Gleason grade	4	4	0.742
Analgesic use (%)	42.8	34.5	0.690
Previous cancer therapy (%)			
Antiandrogen	100	93.1	1.000
Second line anti-hormone agent	85.7	48.2	0.104
Chemotherapy	28.6	31	1.000
Docetaxel	14.2	17.2	1.000
Median serum PSA* (ng/mL)	20.9	5.8	0.122

RP = rapid progression; NRP = non-rapid progression; PSA = prostate-specific antigen *.

## 4. Discussion

Our study of prostate cancer patients found several patients who experienced progression or death within four months of receiving sipuleucel-T, but predictors significantly associated with rapid progression were not identified. Mechanisms for rapid progression of metastatic prostate cancer after sipuleucel-T therapy are not completely understood. Our findings are consistent with several other studies that have found that a majority of patients to progress after receiving sipuleucel-T. The results of our study are also similar to results from previous studies that found prior use of chemotherapy in prostate cancer did not influence progression of the disease after receiving sipuleucel-T [3,4,10]. Chemotherapy such as docetaxel, has shown an overall survival benefit in prostate cancer patients rather than effects on progression. Therefore, there are some thoughts that the effects of chemotherapy in patients who go on to receive sipuleucel-T may not be a significant factor in disease progression [10]. Our findings also indicate that baseline Gleason score may not be a good predictor of progression. The RP group was found to have a lower Gleason score than the NRP group, but that difference was not significant. A possible explanation for this finding is that the Gleason score is a composite score including the primary Gleason grade. The primary Gleason grade describes a greater portion of the histology and aggressiveness of the tumor. In our patients, the RP and NRP groups had similar primary Gleason grades and this factor could possibly contribute to why the overall Gleason scores were not found to be a predictor despite a difference between the two groups. 

Serum PSA has been found to be a risk factor for progression of prostate cancer and studies have indicated that serum PSA indicates burden of disease [11]. Therefore, higher PSA indicates more disease for the immune system to overcome after receiving sipuleucel-T. A recent abstract presented at the American Society of Clinical Oncology (ASCO) found that asymptomatic patients with extensive burden of prostate cancer did not benefit from receiving sipuleucel-T compared to those with a lower burden of disease who received the therapy [12]. These findings about sipuleucel-T therapy and extent of disease burden raise potentially more areas for further research.

In terms of adverse events related to the use of sipuleucel-T, our study found a small incidence of infusion-related reactions that were primarily associated with fevers and chills. This result is also consistent with findings in previous trials. Unlike previous studies, none of our patients experienced cerebral vascular events, but this finding may be due to the small sample size and relatively short follow-up in the study.

Other limitations to this study exist. First, the retrospective nature of this study is a limitation as the data collected is restricted to information available only in the medical record. For example we captured patients who were asymptomatic from pain by examining use of analgesic as a surrogate since baseline pain scores were not available. It was not possible to determine if the use of analgesics were strictly related to pain from prostate cancer or pain not associated with the disease. A major limitation is the relatively small sample size. The study may not have been powered enough to detect a significant difference in factors between the RP and NRP groups. With less than forty patients in this group segregated into 29 patients in the NRP group and seven in the RP group, the size of the group was likely too small to detect a meaningful difference. The small population does not allow any firm conclusions about the differences between them to be drawn.

Additionally, this study also describes a single institution experience with a population that may not reflect that of the general population. Another limitation is that our facility is a referral cancer center and patient follow-up is challenging, especially if a patient chooses to receive only local follow-up near home. Therefore some medical records including lab values may not have been available in our electronic medical record. Furthermore, other potential predictive factors for rapid progression may not have been included in the univariate analysis for this study. Beer *et al*. recently conducted a double-blinded randomized controlled study found a significant increase in PSA doubling time in those using sipuleucel-T over those who did not receive the immunotherapy [13]. Given consistent and timely PSA values reported at the same institution, PSA doubling time would be a potential factor to look at in the future.

Sipuleucel-T is also a relatively new therapy compared to options such as chemotherapy and other anti-hormones. As a result, the length as well as the small number of patients who received sipuleucel-T in this study may not have allowed for adequate analysis of progression. A way to overcome this limitation would be to broaden the time frame to increase the population in the study. This could also potentially allow for examining factors associated with overall survival.

## 5. Conclusions

In conclusion, we found that several patients rapidly progressed despite receiving sipuleucel-T therapy. However we were unable to identify predictors or factors associated with rapid progression within our population. As more information emerges about sipuleucel-T and as newer therapies for the treatment of prostate cancer become available, studies should be conducted in order to help guide its use in clinical practice and to continue to evaluate its benefit in disease progression and overall survival. 
